# Integrated transcriptomic identification and validation reveal key autophagy-associated biomarkers in sleep deprivation

**DOI:** 10.7717/peerj.21426

**Published:** 2026-06-03

**Authors:** Lutong Gan, Zerui You, Wanyue Tan, Simeng Feng, Yixian Cai, Xian Shi, Xia Ma, Jiaqi Yu, Jiyang Pan

**Affiliations:** 1 Sleep Medicine Centre, Department of Psychiatry, The First Affiliated Hospital of Jinan University, Guangzhou, Guangdong, China; 2 Department of Anesthesiology, The Second Affiliated Hospital of Guangzhou Medical University, Guangzhou, Guangdong, China; 3 Nephrology Department, The First Affiliated Hospital of Jinan University, Jinan University, Guangzhou, Guangdong, China

**Keywords:** Sleep deprivation, Insomnia, Autophagy, Single-cell sequencing, ML

## Abstract

**Background:**

Sleep deprivation (SD) is harmful to individuals, but its pathogenesis is not clarified.

**Objective:**

This study seeks to identify SD-linked autophagy genes *via* integrated transcriptomic and experimental validation approaches.

**Methods:**

Primary SD transcriptomic datasets (GSE33302 and GSE9442), derived from murine brain tissue, were retrieved from the Gene Expression Omnibus (GEO) database to identify differentially expressed genes (DEGs). Murine gene symbols were subsequently mapped to their human orthologs to enable downstream bioinformatic analyses and integration with the GeneCards database. The converted DEGs were intersected with autophagy-related genes (ARGs) obtained from GeneCards to identify autophagy-associated DEGs, which were then subjected to functional enrichment analyses. Candidate predictor genes were selected using machine learning (ML) algorithms. Their expression was rigorously validated in both internal and external datasets (GSE9441 and GSE3767), encompassing murine brain tissue and human peripheral blood samples, respectively. In parallel, an SD rat model was established by exposing male Sprague-Dawley rats to continuous sleep deprivation for seven consecutive days. Brain tissues from the prefrontal cortex and hippocampus were harvested, and the expression levels of rat orthologs of the candidate genes were quantified using reverse transcription-quantitative polymerase chain reaction (RT-qPCR). The diagnostic performance of the identified genes was evaluated through nomogram construction and receiver operating characteristic (ROC) curve analysis. In addition, the immune landscape associated with SD was inferred using single-sample gene set enrichment analysis (ssGSEA). The cellular distribution and functional roles of the candidate genes were further explored *via* single-cell RNA sequencing (scRNA-seq; GSE37665) and gene set enrichment analysis (GSEA). Finally, potential therapeutic targets associated with these genes were predicted.

**Results:**

Three significantly dysregulated predictor genes: *CDKN1A*, *HSPA5*, and *NR4A1*, were identified, and a diagnostic model incorporating these genes demonstrated strong predictive performance. Bioinformatic analysis of immune cell infiltration indicated a potential association between the three key predictor genes and modifications in the immune microenvironment. Moreover, single-cell transcriptomic profiling revealed that these genes were preferentially and highly expressed in endothelial cells, glial cells, and neurons, respectively, implying distinct functional roles across different cellular subpopulations.

**Conclusion:**

*CDKN1A, HSPA5,* and *NR4A1* emerge as crucial pathogenic biomarkers and potential therapeutic targets for SD. This study provides novel molecular targets for elucidating the mechanisms underlying SD-induced autophagy modulation, immune response, and neurovascular injury.

## Introduction

Sleep is crucial in maintaining both physiological and psychological well-being. Sufficient sleep is fundamental to overall health and is essential for growth and development, emotional regulation, metabolic waste clearance, oxidative stress (OS) repair, and memory consolidation (Irwin, 2015; Irwin, 2019; Van Cauter et al., 2007). Sleep deprivation(SD) often results from or manifests as sleep disorders and can be caused by various factors like lifestyle choices, occupational stress, environmental influences, and sleep disorders like insomnia and obstructive sleep apnea syndrome. These factors lead to abnormalities in sleep quantity, structure, and/or quality (Krause et al., 2017; Mukherjee et al., 2024; Zhao et al., 2023). SD has increasingly become a significant concern in public health. According to global sleep research data, over 30% of individuals experience sleep disorders, with a subset suffering from short-term or chronic SD (Liu et al., 2016; Watson et al., 2015). The adverse effects (AEs) of insufficient sleep on health are multifaceted, causing fatigue, impaired attention, and cognitive dysfunction, and chronic diseases like cardiovascular disease, metabolic syndrome, depression, and neurodegenerative disorders (Addo et al., 2024; Grandner, 2017; Liew & Aung, 2021; Tempesta et al., 2018). Short-term SD, prolonged sleep restriction, disrupted circadian rhythm, and untreated sleep disorders have a profound and detrimental influence on mental well-being, emotional stability, physical health, as well as public safety (Ramar et al., 2021).

Autophagy refers to a cellular degradation mechanism facilitated by lysosomes, which eliminates superfluous or impaired cellular elements. This process is crucial for maintaining cellular equilibrium, especially in managing reactive oxygen species (ROS), reactive nitrogen species (RNS), intracellular communication, and protein breakdown. Therefore, it ensures a stable balance among the production of organelles, protein creation, and their removal (Bejarano & Cuervo, 2010; Lee, Giordano & Zhang, 2012). Autophagy is primarily classified into three distinct mechanisms, all of which facilitate the degradation of protein substrates within the lysosomal lumen: (1) Autophagy, often termed macroautophagy, is a sequential mechanism characterized by the creation of double-layered vesicles called autophagosomes. These vesicles, once fully developed, merge with lysosomes, enabling the breakdown of their internal materials through the action of acid–based enzymes in a low-pH setting (He & Klionsky, 2009); (2) The process of microautophagy entails the direct engulfment of cytoplasmic materials by lysosomes through membrane invagination, followed by their breakdown *via* enzymes (Chen & Klionsky, 2011); (3) Chaperone-mediated autophagy (CMA) features the specific identification of proteins with a KFERQ-like pentapeptide motif through chaperone proteins. These complexes then attach to LAMP-2A receptors located on the lysosomal membrane, enabling the transport and breakdown of designated proteins inside the lysosome (Mijaljica, Prescott & Devenish, 2011). Dysregulated autophagy has been implicated in neurodegenerative illnesses, autoimmune disorders, inflammation, metabolic dysregulation, and cancer, among others (Luo et al., 2025). Its role in the nervous system is particularly critical, especially in neurons, where non-selective autophagy is key to maintaining neuronal homeostasis (Hara et al., 2006). Autophagy regulates axon guidance, vesicular transport, dendritic spine structure, and pruning, as well as synaptic plasticity by clearing aggregated proteins and maintaining mitochondrial balance (Chen & Klionsky, 2011; Khan, Upadhyay & Hassan, 2024). Impaired autophagy has been suggested as a central mechanism underlying the neurotoxic effects of SD. Recent studies have demonstrated that SD exerts direct effects on neurons by modulating autophagic activity, thereby disrupting neuronal metabolism and homeostasis. These disruptions cause cognitive damage, memory deficits, and depression (Cao et al., 2019; Lin et al., 2025; Ortiz-Vega et al., 2024; Yan et al., 2025). Specifically, the activation of mTOR and AMPK signaling pathways, triggered by SD-induced OS, mitochondrial dysfunction, and protein aggregation, plays a key role in modulating autophagy. These alterations ultimately disrupt neuronal homeostasis, leading to cellular damage, metabolic dysregulation, functional decline, and impaired brain function (Xiong et al., 2024; Zhao et al., 2024). Nevertheless, the molecular mechanisms underlying the role of autophagy in SD remain elusive.

**Figure 1:**
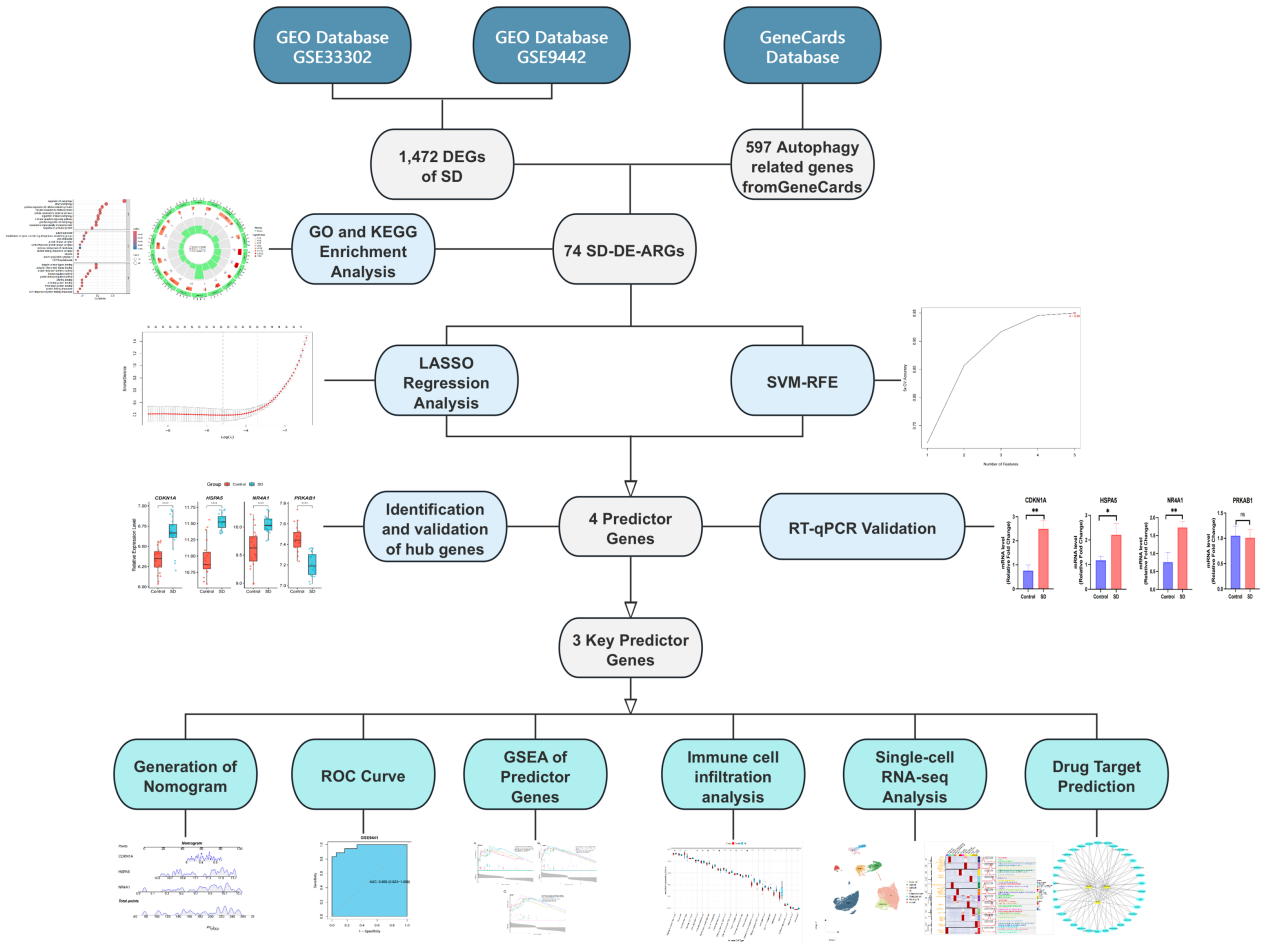
Research flowchart.

In this study, transcriptomic data related to SD were integrated. Key genes linked to SD were identified *via* integrated transcriptomic and experimental validation approaches. Experimental validation confirmed the predictor genes, upon which an SD-related diagnostic model was constructed. The relations of these genes to immune cell infiltration were unraveled, and their cellular localization and immune functions were further elucidated through single-cell analysis. Moreover, potential therapeutic agents targeting these genes were identified. The overall workflow is illustrated in Fig. 1.

## Methods

### Data collection

Three SD datasets were obtained from GEO (http://www.ncbi.nlm.nih.gov/geo/), including GSE9442, GSE33302, GSE9441, and GSE37667, along with a single-cell RNA sequencing (scRNA-seq) dataset, GSE137665 (Jha et al., 2022; Maret et al., 2007; Pellegrino et al., 2012; Vecsey et al., 2012). The GSE9442 dataset comprised 9 control samples and 8 SD samples, whereas GSE33302 included 12 control and 12 SD samples. The GSE9441 dataset, containing nine control and nine SD samples, was excluded from the present analysis because these samples were derived from peripheral tissue (liver). GSE37667 contained nine control and nine SD samples, but nine samples collected after recovery sleep were excluded. The GSE137665 single-cell dataset included three SD samples and three control samples, with three post-recovery sleep samples excluded (Table 1). The GSE9442 and GSE33302 datasets were integrated *via* the removeBatchEffect function from the limma package (ver. 3.60.6). Specifically, the dataset source was defined as the batch effect, while the experimental grouping was included in the design matrix to strictly preserve the biological variance during batch correction. The GSE9441 and GSE37667 datasets were reserved as independent validation sets for assessing the performance of the diagnostic model. Autophagy-related genes (ARGs) were retrieved from the GeneCards database, and those with a Relevance Score greater than 4 were selected for subsequent analysis as highly associated with autophagy.

**Table 1:** Summary of included GEO datasets and samples.

Dataset ID	Platform	Organism	Species/sex	SD paradigm (duration)	Sample size (original)	Sample size (included)	Groups selected	Tissue region	Role in study	Reference
GSE9442	GPL1261	Mus musculus	Male C57BL/6J Mice	Acute SD (6 h, ZT0-ZT6); Gentle handling	17	17	Control (*n* = 9), SD (*n* = 8)	brain tissue	Training set	Maret et al. (2007)
GSE33302	GPL1261	Mus musculus	Male C57BL/6J Mice	Acute SD (5 h, ZT0-ZT5); Gentle handling	24	24	Control (*n* = 12), SD (*n* = 12)	brain tissue	Training set	Vecsey et al. (2012)
GSE9441	GPL1261	Mus musculus	Male C57BL/6J Mice	Acute SD (6 h, ZT0-ZT6); Gentle handling	36	18	Control (*n* = 9), SD (*n* = 9)	brain tissue	Validation set	Maret et al. (2007)
GSE37667	GPL570	Homo sapiens	Homo sapiens (Male)	Total SD (>24 h wakefulness)	27	18	Control (*n* = 9), SD (*n* = 9)	Whole Blood	Validation set	Pellegrino et al. (2012)
GSE137665	GPL21103	Mus musculus	Male C57BL/6J Mice	Acute SD (12 h, ZT0-ZT12); Automated sweeper + Gentle handling	9	6	Control (*n* = 3), SD (*n* = 3)	brain tissue	scRNA-seq Analysis	Jha et al. (2022)

### Identification of differentially expressed ARGs linked to SD (SD-DE-ARGs)

Differentially expressed genes (DEGs) linked to SD were identified utilizing “limma” (ver. 3.60.6) *via* differential expression analysis, with a threshold of adjusted *p* < 0.05 for gene selection. Subsequently, the DEGs were intersected with 597 ARGs to obtain SD-DE-ARGs. Finally, visualization was conducted using the “ggplot2” (ver. 3.5.1) and “ggvenn” (ver. 0.1.16) packages.

### GO and KEGG functional enrichment analysis

Gene Ontology (GO) and Kyoto Encyclopedia of Genes and Genomes (KEGG) enrichment analyses were carried out on SD-DE-ARGs *via* the “clusterProfiler” package (ver. 4.4.4) to explore their biological functions. A false discovery rate (FDR)-adjusted *p* < 0.05 denoted statistical significance. Results were visualized *via* the “circlize” package (ver. 0.4.16) and “ggplot2” package (ver. 3.5.1).

### Screening of predictor genes for SD

ML algorithms, like Least Absolute Shrinkage and Selection Operator (LASSO) and Support Vector Machine Recursive Feature Elimination (SVM-RFE), were leveraged for SD-DE-ARGs screening. The LASSO algorithm determines the variables by analyzing the minimum classification error *λ*, which is utilized to select feature variables and develop the optimal classification model. Five-fold cross-validation and regression analysis with tuning/privacy parameters were enabled by the “glmnet” package (ver. 4.1.8) for feature selection, model construction, and handling high-dimensional data (Qiu et al., 2024). SVM-RFE, an approach for feature selection utilizing support vector machines, was conducted using five-fold cross-validation through the “e1071” package (ver. 1.7.16). This method recursively eliminates the least important features to select the most representative subset (Peng et al., 2024). Finally, the intersection of the genes selected by both ML algorithms was defined as the predictor genes for SD. Visualization was performed using the “ggvenn” (ver. 0.1.16) and “ggplot2” (ver. 3.5.1) packages.

### Reverse Transcription-Quantitative Polymerase Chain Reaction (RT-qPCR)

Male Sprague-Dawley rats, eight weeks of age and weighing 200–220 g at baseline, were obtained from Guangdong GemPharmatech Co., Ltd. All experimental procedures were conducted in accordance with the guidelines approved by the Institutional Animal Care and Use Committee (IACUC) of the First Affiliated Hospital of Jinan University (Approval No. IACUC-2025080107). Each SD and control group consisted of three randomly assigned rats, determined based on the principle of minimum sample size (*n* = 3). Randomization was performed to reduce potential selection bias. All cages were maintained on the same rack under identical environmental conditions to control for spatial confounders. However, due to operational constraints, euthanasia and subsequent tissue processing were conducted in a fixed sequence, with the SD group processed before controls. Investigators were not blinded to group allocation during outcome assessment. During acclimation, rats were housed in standard polycarbonate cages (45 cm × 30 cm × 20 cm) at a density of three rats per cage, with bedding replaced twice weekly. The animal facility maintained a 12:12 h light-dark cycle, a temperature of 22 ± 2 °C, and a relative humidity of 50 ± 5%. Environmental enrichment, including PVC tunnels and wooden blocks, was provided and renewed weekly. Standard rodent chow and water were available ad libitum. Rats were acclimated for two weeks before experimentation. The SD model was established according to the method described by Kaushal et al. (2012). The apparatus consisted of a rotatable deprivation bar traversing the cage floor intermittently, compelling the rats to move to avoid contact. When activated, the deprivation bar rotated at ten revolutions per minute, alternating between clockwise 1.5 turns, counterclockwise 1.5 turns, and clockwise 1.5 turns. At the onset of the experiment, rats in both groups were allowed unrestricted movement, feeding, and drinking within the SD chamber. For the SD cohort, the apparatus was activated daily from the fourth hour of the light phase to the twelfth hour of the dark phase for seven consecutive days, restricting sleep to approximately four hours per day. In contrast, the apparatus remained inactive throughout the experimental period in the control cohort. At the end of the deprivation protocol, rats were deeply anesthetized by intraperitoneal injection of 2% pentobarbital sodium (50 mg/kg). Adequate anesthetic depth was confirmed by the absence of pedal reflexes. Euthanasia was performed *via* cervical dislocation, and death was confirmed by cessation of heartbeat and respiration. The brain was immediately removed and placed on ice-cold PBS-soaked filter paper. For hippocampal dissection, the overlying cortex was carefully peeled away using fine forceps, and the hippocampus was bilaterally excised with removal of the alveus and fimbria. The entire hippocampal formation, including the dentate gyrus and CA1-CA3 subfields, was collected. For prefrontal cortex dissection, the medial prefrontal cortex was targeted based on coordinates from the Paxinos and Watson Rat Brain Atlas, approximately Bregma +4.70 mm to +2.70 mm (Paxinos & Watson, 2006). Coronal slices (∼2 mm) were sectioned using a chilled razor blade. Minor inclusion of the corpus callosum was unavoidable but consistent across all samples. Dissected tissues were immediately snap-frozen in liquid nitrogen and stored at −80 °C. mRNA expression of the predictor genes in these tissues was quantified by RT-qPCR. Animals exhibiting >20% body weight loss, severe lethargy, unhealed wounds, or signs of prolonged distress would have been euthanized before the planned endpoint. Nevertheless, no animals met these humane endpoint criteria. All rats were monitored daily to ensure welfare. Total RNA was extracted from brain tissues using TRIzol reagent (Invitrogen, USA), and RNA concentration was determined spectrophotometrically. Complementary DNA (cDNA) was synthesized using the EntiLink™ 1st Strand cDNA Synthesis Kit (ELK Biotechnology, EQ003) according to the manufacturer’s instructions. Reverse transcription was performed at 25 °C for 5 min, 42 °C for 30 min, and 85 °C for 5 min.

Quantitative analysis of mRNA expression was carried out using the EnTurbo™ SYBR Green PCR SuperMix Kit (ELK Biotechnology, EQ001) on a StepOne™ Real-Time PCR System (Life Technologies, Carlsbad, CA, USA).

The relative gene expression levels were calculated using the 2^−ΔΔCt^ method, with GAPDH serving as the internal reference. The primer sequences used in the RT-qPCR assays are listed in Table S1.

### Identification and validation of predictor genes

Expression differences of the predictor genes between the two groups were assessed in the training datasets (GSE9442 and GSE33302). Two independent datasets, encompassing both target brain and blood samples (GSE9441 and GSE37667), were employed for validation. Results were visualized *via* the “ggstatsplot” package (ver. 0.13.0). A diagnostic model for SD was constructed based on multivariate logistic regression using the identified predictor genes. Calibration curves were generated for the diagnostic model. Receiver operating characteristic (ROC) analysis was carried out *via* “pROC” (ver. 1.18.5), and the diagnostic performance of the model was examined *via* the area under the curve (AUC) with its 95% confidence interval (95% CI).

### GSEA functional enrichment analysis

The functional relevance of the predictor genes was investigated using gene set enrichment analysis (GSEA). Briefly, Spearman correlation coefficients were computed between each predictor gene and all other genes within the normalized expression matrix. Genes were subsequently ranked in descending order according to these correlation coefficients to generate a pre-ranked gene list. GSEA was then performed using the clusterProfiler R package (ver. 4.12.6). The human-specific GO gene set from the Molecular Signatures Database (MSigDB; “c5.go.v2024.1.Hs.symbols.gmt”) was used as the reference. Pathways with a nominal *P* value < 0.05 were considered significantly enriched.

### Immune infiltration analysis

To assess SD-induced alterations in the immune microenvironment, single-sample gene set enrichment analysis (ssGSEA) was conducted using the GSVA package in R. The fully normalized, transcriptome-wide expression matrix from the merged SD dataset served as input. A curated gene signature encompassing 28 immune cell subpopulations was applied to estimate the relative infiltration abundance of each immune cell type in individual samples (Charoentong et al., 2017). Pearson correlation analysis was subsequently performed to evaluate associations between immune cell infiltration scores and the expression levels of the predictor genes. The results were visualized using the ggplot2 R package (ver. 3.5.1).

### scRNA-seq

scRNA-seq data obtained from GEO (GSE137665) were preprocessed and annotated using the Seurat package (ver. 5.3.0) (Jha et al., 2022). Quality control criteria included detection of 300–2,000 genes per cell and a mitochondrial gene proportion below 10%. Cells passing quality control were normalized and scaled. Highly variable genes were identified *via* the FindVariableFeatures function. Linear dimensionality reduction was performed *via* principal component analysis (PCA), followed by nonlinear reduction using UMAP (dims = 1:20). Unsupervised clustering was conducted using the Louvain algorithm with a resolution parameter of 0.5, selected based on clustering stability and biological consistency. Cell type annotation was performed manually based on established marker genes. Differential expression analysis was performed using the FindAllMarkers function (logfc.threshold = 0.25, min.pct = 0.1), and the top 30 marker genes per cell type were selected for functional enrichment analysis. Cell type-specific marker genes and target gene expression were visualized using violin plots and dot plots.

### Drug prediction based on key predictor gene targets

To identify potential candidate drugs targeting key predictor genes, a reverse drug screening was performed using the Drug Signatures Database (DSigDB, Version 1.0; https://dsigdb.tanlab.org/DSigDBv1.0/). DSigDB integrates drug-induced gene expression profiles from multiple public databases, including Connectivity Map, LINCS, and DrugBank. Potential drugs and their corresponding predictor gene targets were visualized as interaction networks *via* Cytoscape (Otasek et al., 2019).

### Data analysis

All data analyses were enabled by R (ver. 4.4.1). To evaluate statistical differences across groups, either the Wilcoxon test or the *t*-test was applied based on data distribution characteristics. Correlation analyses were conducted using Pearson correlation. Each statistical test was two-sided, with *p* < 0.05 denoting statistical significance.

### Ethical approval and consent to participate

This animal study gained the approval of the Animal Ethics Committee of the First Affiliated Hospital of Jinan University (Approval No.: IACUC-2025080107) and followed the ARRIVE 2.0 guidelines. All experiments strictly adhered to the 3R principle, minimizing animal use and suffering while ensuring robust statistical outcomes. All experimental procedures and handling protocols were conducted as per the IACUC of the First Affiliated Hospital of Jinan University.

## Results

### Identification of DEGs linked to autophagy

**Figure 2:**
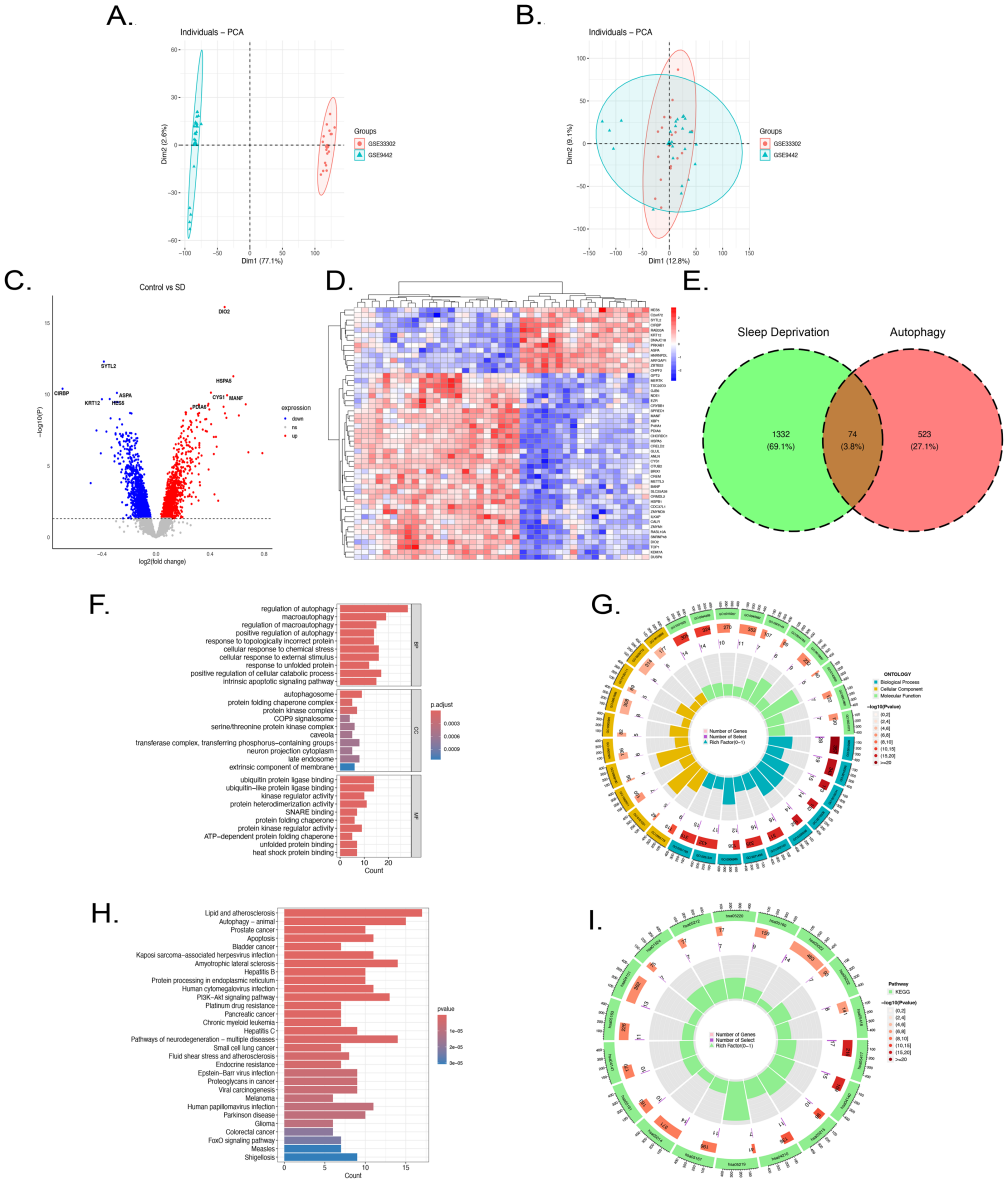
SD-DE-ARGs Identification and GO and KEGG FEA of SD-DE-ARGs. (A–B) PCA plots before and after the removal of batch effects for GSE9442 and GSE33302 datasets. (C) Volcano plot of DEGs identified through differential analysis. (D) Clustering heatmap of the expression levels of the top 50 DEGs. (E) Venn diagram illustrating the intersection between DEGs in SD and ARGs. (F–G) GO FEA, including BP, CC, and MF. (H–I) KEGG pathway enrichment analysis.

The GSE9442 and GSE33302 datasets were merged, and batch effect removal was performed, resulting in 20 SD samples and 21 control samples (Figs. 2A–2B). Subsequently, the differential analysis identified 702 upregulated genes and 770 downregulated genes, with heatmaps and volcano plots presented in Figs. 2C–2D. Finally, ARGs were retrieved from GeneCard, and genes with a correlation coefficient greater than 4, strongly linked to autophagy, were selected. The intersection of DEGs and high ARGs yielded 74 SD-DE-ARGs (Fig. 2E).

### Functional enrichment analysis

To elucidate the systemic biological roles of SD-DE-ARGs beyond their canonical functions, GO and KEGG enrichment analyses were performed. In the Biological Process (BP) category, while the genes were primarily clustered in autophagy and macroautophagy regulation, the analysis also revealed their specific involvement in stress adaptation mechanisms, including cellular responses to chemical stress and misfolded proteins. This indicates that SD-DE-ARGs act as critical mediators of cellular quality control under pathological stress. Cellular Component (CC) analysis showed that, in addition to autophagosomes, the gene products were significantly localized to protein kinase complexes and protein-folding chaperone complexes, highlighting their roles in signal transduction and protein stability. Molecular Function (MF) analysis further revealed prominent activities in ubiquitin ligase binding, kinase regulator activity, and SNARE binding, emphasizing their regulatory complexity in protein degradation and vesicle fusion. KEGG pathway analysis uncovered extensive signaling crosstalk and pleiotropic effects. Beyond the expected enrichment in the Autophagy (animal) pathway, SD-DE-ARGs were significantly associated with non-autophagic pathways, including Apoptosis (reflecting regulation of cell death), Lipid metabolism and atherosclerosis (suggesting metabolic implications), and disease-specific pathways such as Prostate cancer and Amyotrophic lateral sclerosis (ALS). These results (Figs. 2F–2I) indicate that SD-DE-ARGs form a complex signaling network connecting autophagy with metabolic regulation and tumorigenesis.

### Screening of predictor genes linked to SD

**Figure 3:**
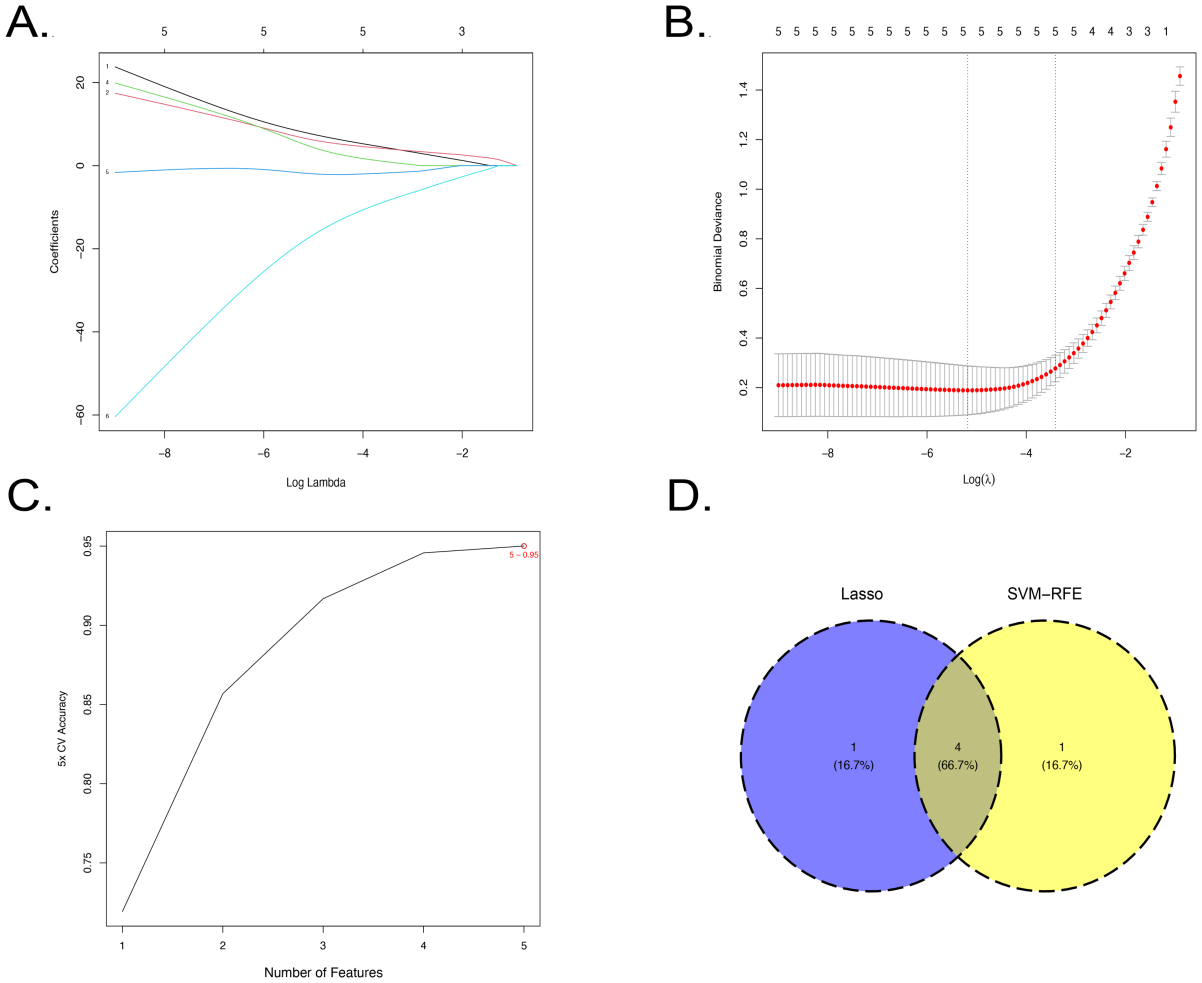
The process of screening predictor genes linked to SD. (A–B) Five genes with diagnosis values were noted through LASSO regression analysis within the SD-DE-ARGs. The optimal regularization parameter (*λ*) was selected for cross-validation. (J) Five genes with diagnostic values were identified within the SD-DE-ARGs using the SVM-RFE algorithm. (D) A Venn diagram of the predictor genes was generated by the intersection of the LASSO and SVM-RFE algorithms.

LASSO and SVM-RFE algorithms were employed to further screen the 74 SD-DE-ARGs. Using the LASSO regression algorithm, five diagnostic genes were identified from the SD-DE-ARGs: *CDKN1A, HSPA5, NR4A1, OFD1,* and *PRKAB1* (Figs. 3A–3B). Through the application of the SVM-RFE algorithm, five potential diagnostic biomarkers were pinpointed, including *PRKAB1, CDKN1A, NR4A1, HSPA5,* and *HSPB1* (Fig. 3C). Subsequently, after integrating the feature genes derived from both analytical approaches, four core regulatory genes were identified: *CDKN1A, HSPA5, NR4A1,* and *PRKAB1* (Fig. 3D).

### Differential expression of predictor genes and RT-qPCR validation

**Figure 4:**
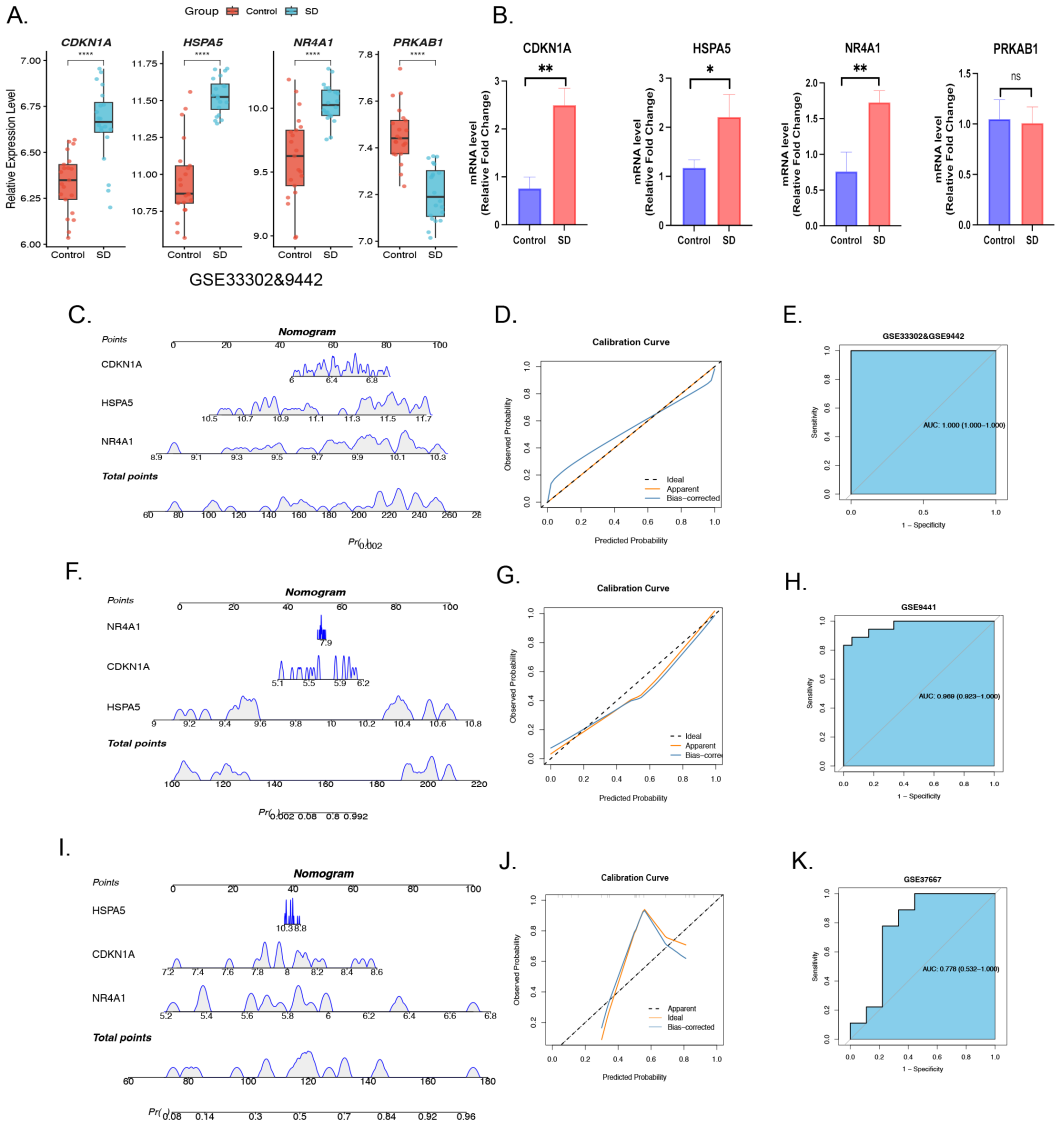
Differential expression and diagnostic value of key predictor genes: RT-qPCR and external cohort validation. (A) Differential expression of CDKN1A, HSPA5, NR4A1, and PRKAB1 between the SD group and the control group in the training set. (B) Validation of SD-related predictor genes by RT-qPCR. (C) Nomogram for predicting SD based on key predictor genes. (D) Calibration curve showing the clinical utility of the nomogram. (E) ROC curve of the predictor gene-based diagnostic model in the training set. (F) Nomogram for predicting SD in the validation set (GSE9441) based on key predictor genes. (G) Calibration curve demonstrating the clinical utility of the nomogram in the validation set. (H) ROC curve of the predictor gene-based diagnostic model in the validation set. (I) Nomogram for predicting SD in the validation set (GSE37667) based on key predictor genes. (J) Calibration curve demonstrating the clinical utility of the nomogram in the validation set. (K) ROC curve of the predictor gene-based diagnostic model in the validation set. **** indicates *p* < 0.0001; *** indicates *p* < 0.001; ** indicates *p* < 0.01; * indicates *p* < 0.05.

The expression of four predictor genes was investigated across the two groups. In the training sets (GSE33302 & GSE9442), *CDKN1A, HSPA5,* and *NR4A1* were significantly upregulated in the SD cohort, whereas *PRKAB1* was notably downregulated (Fig. 4A). To further validate the differential expression of predictor genes, RT-qPCR was performed on three Sprague-Dawley rats and three normal rats. Compared with the healthy control group, *CDKN1A* (*p* < 0.01), *HSPA5* (*p* < 0.05), and *NR4A1* (*p* < 0.01) were significantly upregulated in the SD group, whereas *PRKAB11* showed an insignificant difference across the two cohorts (Fig. 4B, Tables S2–S3). Ultimately, based on the significant upregulation confirmed by RT-qPCR and the lack of significance for *PRKAB1*, we established *CDKN1A, HSPA5,* and *NR4A1* as the final panel of key predictor genes (Fig. 4B).

### Diagnostic value of key predictor genes and external cohort validation

To evaluate the diagnostic efficacy of the three identified ARGs (*NR4A1, HSPA5,* and *CDKN1A*), a diagnostic nomogram was constructed for SD risk assessment (Fig. 4C). The nomogram assigns weighted points according to the regression coefficients of each gene, with *NR4A1* contributing the most to the risk score (expression range: 8.9–10.3), followed by *HSPA5* (10.5–11.7) and *CDKN1A* (6.0–6.8). In the training cohort (GSE33302 and GSE9442), the calibration curve demonstrated strong concordance between predicted probabilities and observed outcomes, with minimal deviation from the ideal diagonal line (Fig. 4D). ROC analysis revealed an AUC of 1.000 (95% CI [1.000–1.000]), indicating excellent discriminatory ability of the three-gene signature within the training dataset (Fig. 4E). External validation using two independent GEO datasets confirmed the generalizability of the diagnostic model. In the first validation cohort (GSE9441), the nomogram maintained robust predictive performance, with an AUC of 0.969 (95% CI [0.923–1.000]), and the calibration curve showed good agreement between predicted and observed probabilities (Figs. 4F–4H). In the second validation cohort (GSE37667), although performance slightly declined relative to the training and first validation sets, the model still exhibited satisfactory discrimination, with an AUC of 0.778 (95% CI [0.532–1.000]). The calibration curve demonstrated reasonable consistency, despite some deviation from the ideal line in the high-probability region (Figs. 4I–4K).

### GSEA functional enrichment analysis

**Figure 5:**
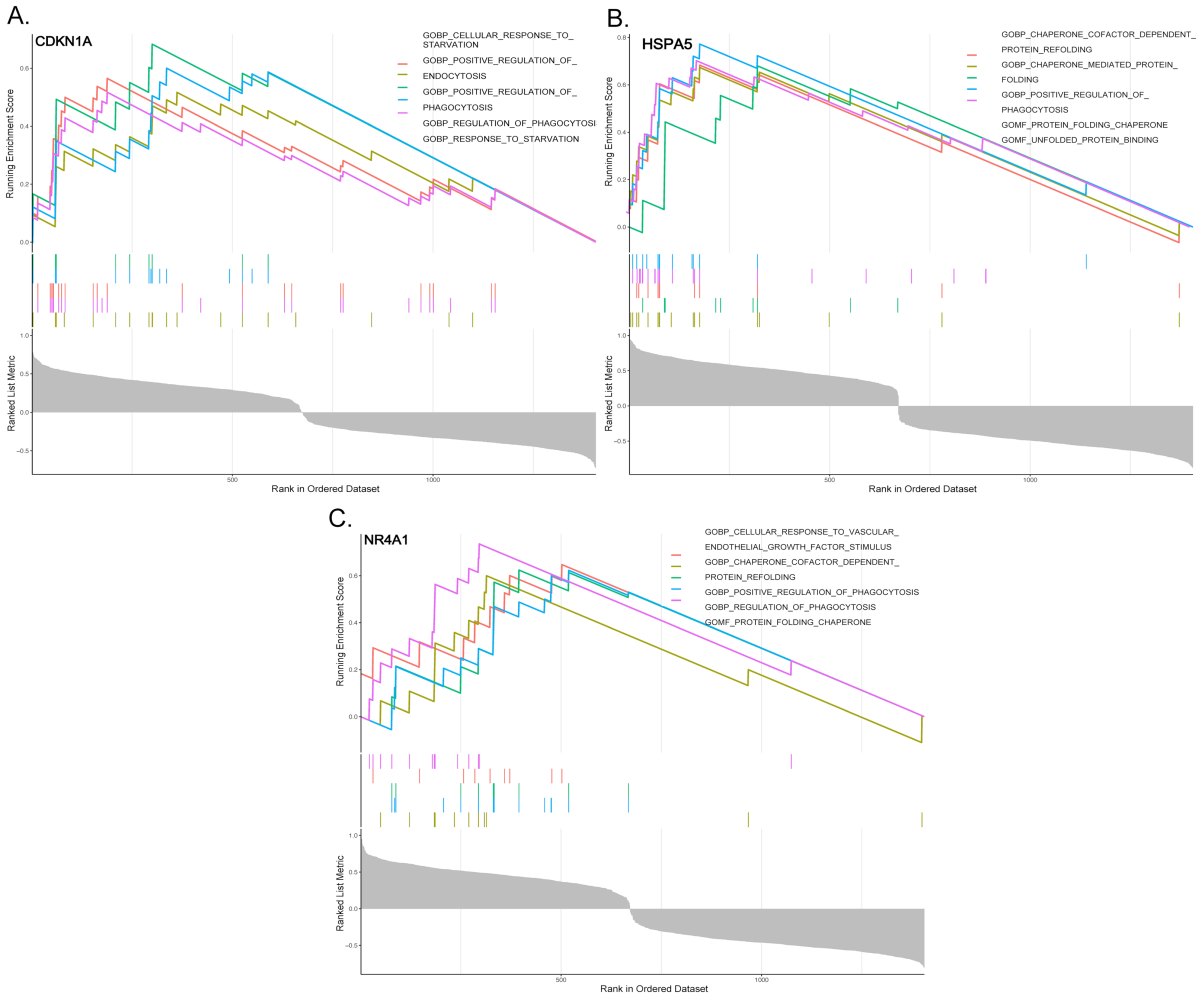
Differential expression of predictor genes in the validation set. (A–D) The differential expression of CDKN1A, HSPA5, and NR4A1 across the SD and control groups in the validation set.

To unravel the biological roles of the predictor genes, GSEA was performed. The findings indicated that these pivotal genes are likely to be significantly involved in the pathological processes of SD by regulating BP, such as endocytosis, positive regulation of phagocytosis, assisting protein folding, and responding to vascular endothelial growth factor signaling pathways (Figs. 5A–5C).

### Immune cell infiltration analysis

**Figure 6:**
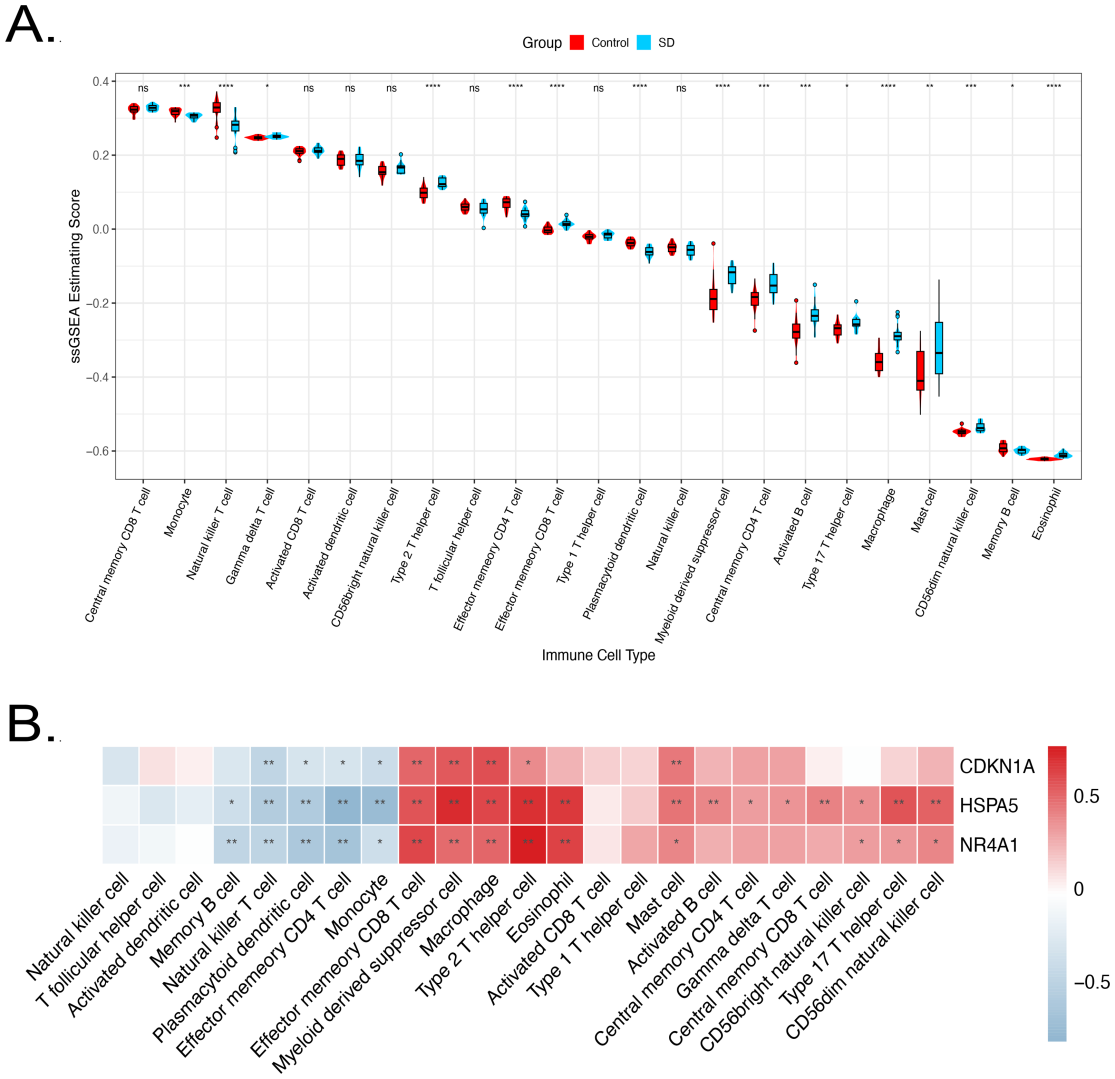
Immune cell infiltration analysis of predictor genes. (A) Correlation between predictor genes and immune cells. (B) Differences in infiltration of 28 immune cell types between the SD and control groups. ^∗^*p* < 0.05, ^∗∗^*p* < 0.01, ^∗∗∗^*p* < 0.001.

Correlation analysis of three key predictor genes with immune cells revealed that *NR4A1* expression was positively related to effector memory CD8^+^ T cells, myeloid-derived suppressor cells, and macrophages, but negatively linked to effector memory CD4^+^ T and plasmacytoid dendritic cells. *HSPA5* showed broad positive correlations with multiple immune cells and a negative relation to effector memory CD4^+^ T and memory B cells. *CDKN1A* was positively related to effector memory CD8^+^ T cells, but negatively correlated with effector memory CD4^+^ T cells. Therefore, the predictor genes possibly play distinct roles in immune regulation (Fig. 6A).

To characterize the global immune landscape in SD, the relative abundance of the 28 immune cell types was compared between SD and control cohorts. Relative to controls, monocytes, natural killer T cells, CD4^+^ T cells, plasmacytoid dendritic cells, and memory B cells were decreased in the SD group. In contrast, type 2 helper T cells, CD8^+^ T cells, myeloid-derived suppressor cells, central memory CD4^+^ T cells, activated B cells, and macrophages were significantly increased (Fig. 6B).

### Expression patterns of predictor genes in single cells

**Figure 7:**
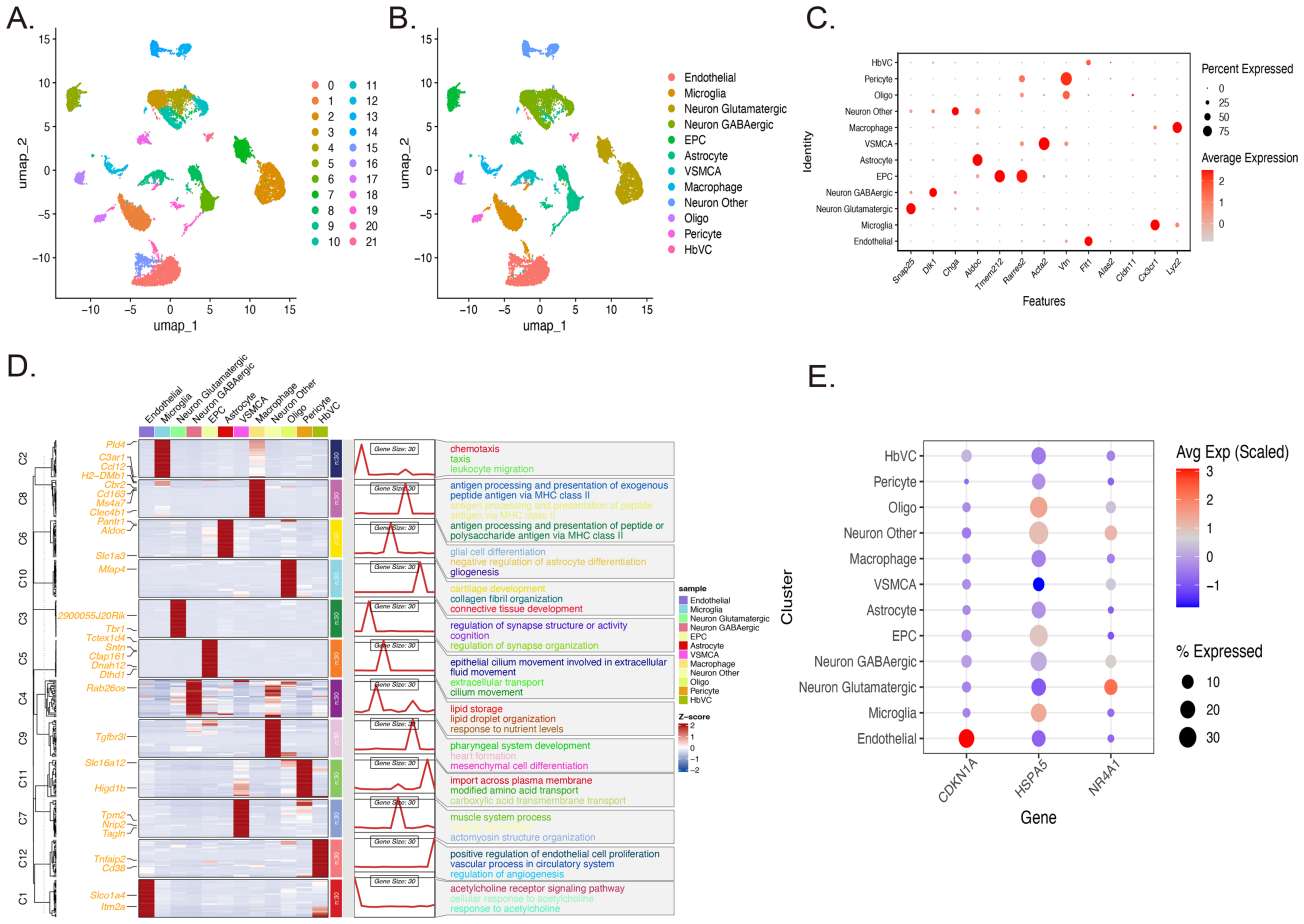
Single-cell transcriptomic landscape and cell-type-specific expression profiles of key predictor genes. (A-B) Identification and visualization of cell populations. (A) UMAP plot from the GSE137665 dataset, revealing 22 distinct transcriptional clusters. (B) UMAP plot showing the annotation of 12 major cell types based on canonical markers, with endothelial cells and glutamatergic neurons constituting the predominant populations. (C) Validation of cell type identities. (D) Functional characterization *via* GO enrichment analysis. (E) Cell-type-specific expression of key predictor genes. Expression patterns of CDKN1A, HSPA5, and NR4A1 across all identified clusters.

scRNA-seq data from the SD dataset (GSE137665) were processed using a standardized workflow and analyzed with the Seurat package. Quality control metrics confirmed high data fidelity, with a median of ∼500 genes detected per cell and a mitochondrial gene proportion of ∼4% (Fig. S1). Unsupervised clustering *via* UMAP dimensionality reduction identified 22 transcriptionally distinct clusters, which were annotated into 12 major cell types based on canonical marker gene expression (Figs. 7A–7B). The cellular composition was dominated by endothelial cells and glutamatergic neurons, occupying the lower-left and right regions of the UMAP embedding, respectively.

Cell type identities were validated through established marker genes (Fig. 7C). Endothelial cells specifically expressed *Flt1* and *Cldn5*; pericytes and vascular smooth muscle cells expressed *Acta2* and *Vtn*; astrocytes expressed *Aldoc* and *Slc1a3*; oligodendrocytes expressed *Cldn11* and *Mbp*; glutamatergic neurons expressed *Snap25* and *Slc17a7*; GABAergic neurons expressed *Dlk1* and *Gad1*; microglia expressed *Cx3cr1* and *P2ry12*; and macrophages expressed *Lyz2* and *Cd14*.

GO functional enrichment analysis revealed cell type-specific biological signatures (Fig. 7D). Microglia were enriched in chemotaxis, leukocyte migration, and antigen processing and presentation *via* MHC class II pathways, consistent with their role as CNS immune sentinels. Macrophages similarly displayed enrichment in antigen presentation pathways but exhibited distinct transcriptional profiles reflecting their peripheral origin. Astrocytes were enriched in glial cell differentiation, gliogenesis, and regulation of synapse structure, highlighting neurosupportive functions. Oligodendrocytes showed enrichment in cartilage development and collagen fibril organization, consistent with myelination roles. Glutamatergic neurons were enriched in synaptic structure regulation, cognition, and cilium movement pathways. Endothelial cells exhibited enrichment in vascular endothelial proliferation regulation, angiogenesis, and acetylcholine receptor signaling, reflecting their angiogenic capacity and neurovascular coupling functions.

Notably, the three key predictor genes, *CDKN1A*, *HSPA5*, and *NR4A1*, displayed highly selective, cell type-specific expression patterns (Fig. 7E). *CDKN1A* demonstrated the most stringent specificity, with robust expression restricted to endothelial cells and negligible expression in all other cell types, including microglia, macrophages, and other immune populations, suggesting a specialized role in vascular homeostasis and endothelial stress responses under SD. *HSPA5* exhibited a broader but distinct pattern, with the highest expression in endothelial progenitor cells (EPCs) and moderate expression in oligodendrocytes, neurons, and astrocytes, while remaining absent in microglia and macrophages. *NR4A1* was predominantly expressed in neuronal lineages, peaking in glutamatergic neurons and showing moderate expression in GABAergic neurons and other neuronal subtypes, while remaining virtually undetectable in non-neuronal populations, including immune cells.

### Prediction of targeted drugs

The three human predictor genes (*CDKN1A, HSPA5,* and *NR4A1*) were queried against the Enrichr platform using the DSigDB database to identify potential therapeutic compounds. Drugs with adjusted *P* < 0.01 were retained, yielding 35 candidate compounds and small molecules targeting these genes, which may represent potential therapeutic strategies for mitigating the adverse effects of SD (Fig. S2, Table S4).

## Discussion

SD has recently emerged as a common phenomenon in modern life, with both chronic and acute SD having profound effects on physiological and psychological health. Research indicates that SD possibly leads to widespread cellular stress responses like OS, endoplasmic reticulum (ER) stress, and mitochondrial dysfunction. These stress responses trigger metabolic disturbances, protein homeostasis imbalances, and organelle damage, which possibly in turn lead to neuronal dysfunction and even apoptosis (Grandner, 2017). To counteract these damages, cells activate a range of protective mechanisms, with autophagy being considered a critical defensive pathway. However, the activation of autophagy possibly has dual effects. On one hand, autophagy helps alleviate OS and ER stress by clearing damaged mitochondria and misfolded proteins, thus maintaining cellular function and promoting cell survival. On the other hand, excessive or dysregulated autophagy possibly exacerbates cellular damage and even induces cell death (Lee, Giordano & Zhang, 2012; Wang et al., 2024). Autophagy has a critical role in SD, yet the exact mechanisms by which autophagy contributes to SD are unclear.

In this study, 74 SD-related differentially expressed ARGs (SD-DE-ARGs) were identified. Functional enrichment analysis highlighted pathways involving autophagosome regulation and lipid metabolism, consistent with previous evidence that SD contributes to neurological and cardiovascular pathologies *via* autophagy modulation (Yan et al., 2025; Zhao et al., 2024). Through machine learning (ML) algorithms and experimental validation in a rat model, three key predictor genes, *CDKN1A, HSPA5,* and *NR4A1,* were pinpointed as significantly upregulated under SD conditions. Although *PRKAB1* did not reach statistical significance in our cohort, likely due to limited sample size, the robust elevation of the other three genes suggests their critical roles as molecular effectors in the SD stress response.

The systemic impact of SD extends beyond cellular stress to immune regulation. ssGSEA revealed a distinct immune landscape in the SD group, characterized by reductions in monocytes, natural killer T (NKT) cells, effector memory CD4^+^ T cells, and plasmacytoid dendritic cells, indicative of immune suppression or exhaustion within the tissue microenvironment. Notably, expression of *CDKN1A, HSPA5,* and *NR4A1* negatively correlated with these immune cell populations. A conventional interpretation of bulk RNA-seq data might suggest that these genes are expressed by the immune cells themselves, marking a transition to a “functionally suppressed state” (Al-Rashed et al., 2025). However, the ssGSEA algorithm infers immune cell proportions from bulk transcriptomic signatures but cannot resolve the cellular source of gene expression, specifically, whether these predictor genes originate from the infiltrating immune cells or the resident tissue compartments.

To resolve the cellular origin of stress signals and elucidate the connection between autophagy dysfunction and immune alterations, scRNA-seq analysis was integrated. Single-cell profiling revealed a pronounced “Neurovascular Unit (NVU)-centric” distribution pattern. *CDKN1A* exhibited specificity for endothelial cells, *HSPA5* was highly enriched in EPCs and glial cells, serving as a vascular-associated stress marker, and *NR4A1* was selectively expressed in glutamatergic neurons.

The apparent discrepancy between ssGSEA results and stress gene expression can be reconciled through a neurovascular unit model supported by emerging evidence. The NVU concept, originally proposed by Iadecola (2004) and subsequently refined, emphasizes the integrated function of neurons, glia, and vascular cells in maintaining brain homeostasis (Iadecola, 2004; Iadecola, 2017). NVU is particularly vulnerable to SD. Yang et al. (2026) demonstrated that SD triggers early and specific upregulation of TRPM4 in hippocampal endothelial cells, inducing blood–brain barrier (BBB) disruption characterized by tight junction loss and perivascular edema, subsequently activating microglial TLR4-NF-κB signaling. Similarly, systematic reviews have confirmed that SD impairs endothelial function through inflammation, OS, and autonomic dysregulation, with elevated systemic inflammatory markers accompanying endothelial dysfunction (Holmer et al., 2021). Single-cell data extend these findings by pinpointing stress signatures in specific NVU compartments that precede and orchestrate immune alterations.

CDKN1A (encoding *p21*) likely mediates SD-induced cerebrovascular dysfunction rather than directly modulating immune responses. CDKN1A is a central regulator of cell cycle progression, playing essential roles in cellular proliferation, differentiation, stress responses, and programmed cell death (Yosef et al., 2017). Beyond cell cycle regulation, CDKN1A regulates autophagy *via* multiple pathways, including p38 MAPK, mTOR, and p53 interactions (Beyfuss & Hood, 2018; Vucicevic et al., 2014; Xie et al., 2012; Yue et al., 2023). Critically, emerging evidence demonstrates that endothelial p21 specifically, not immune cell p21, drives chronic inflammation and tissue dysfunction. Levi et al. (2023) showed that p21 in epithelial and endothelial cells, but not hematopoietic immune cells, mediates inflammatory responses following chronic LPS exposure. Endothelial-specific p21 knockout reduced senescent CDKN1A upregulation and promoted vascular senescence and BBB disruption, creating a permissive environment for secondary immune cell infiltration and microglial activation (Qin et al., 2023; Tan, Zhu & Hashimoto, 2024; Yue et al., 2023). This barrier dysfunction represents the primary trigger altering the immune milieu, explaining ssGSEA patterns without requiring immune cell-autonomous gene expression.

The enrichment of *HSPA5* in EPCs highlights the burden of ER stress on vascular regeneration. *HSPA5* encodes GRP78/BiP, a master regulator of the unfolded protein response (UPR) that supports protein folding, maintains ER homeostasis, and manages cellular stress (Mai et al., 2025). During ER stress, HSPA5 inhibits mTOR activity *via* IRE1 signaling, indirectly promoting autophagy, and interacts with ATF4, the ATG pathway, and Bcl-2 family members to enhance clearance of damaged materials (Jiang et al., 2022; Liang et al., 2020; Mu et al., 2019). EPC-specific *HSPA5* expression is functionally significant: EPCs are crucial for microcirculation maintenance and vascular repair, and their stressed state implies that SD impairs cerebral vascular regenerative capacity (Cerezo & Rocchi, 2017). This ER stress-mediated autophagy dysregulation correlates with broader metabolic disturbances observed in SD, including impaired cerebrovascular reactivity and perfusion deficits (Hao, Song & Zhang, 2023; Kawano et al., 2023; Li et al., 2024). The absence of *HSPA5* in microglia and macrophages further supports an NVU-centric model, indicating that ER stress in SD originates primarily in vascular compartments.

*NR4A1* expression restricted to glutamatergic neurons positions it as a mediator of excitotoxic stress in SD. NR4A1 (Nur77) is an orphan nuclear receptor and immediate-early gene induced by neuronal activity, OS, and calcium influx (Mullican et al., 2007; Won & Hwang, 2016). Its upregulation likely represents a neuroprotective attempt to counteract the metabolic burden caused by hyperexcitability during prolonged wakefulness.

NR4A1 serves as a molecular bridge connecting mitochondrial function, apoptosis, and autophagy in CNS-related diseases (Zheng et al., 2022). Nur77 is induced by excitotoxicity and OS in neurons, with its subcellular localization determining survival *versus* apoptotic outcomes (Wang et al., 2025). In SD, sustained glutamatergic hyperactivity induces calcium overload and mitochondrial stress. The selective expression of *NR4A1* in glutamatergic, but not GABAergic, neurons suggests circuit-specific vulnerability to SD-induced autophagy dysfunction. Moreover, neuron-derived NR4A1 signals may influence microglia through paracrine mechanisms, shaping immune tone without requiring neuronal immune gene expression.

Therefore, SD may cause functional impairment of the NVU, leaving residual cells in a highly stressed state with features of senescence, ER stress, or excitotoxic injury. Although these cells remain present and express core stress genes, their physiological functions may be substantially compromised. This NVU-centric model offers a framework for targeted therapeutic strategies, such as endothelial protection, modulation of ER stress in vascular progenitors, or prevention of excitotoxicity in glutamatergic neurons, rather than broad immunosuppression. The three predictor genes were submitted to the Enrichr database, and associated drugs and targets were screened using DSigDB. Compounds with adjusted *p*-values < 0.05 were selected, identifying 35 drugs and targets as potential therapeutic interventions for mitigating SD-induced AEs.

There are several limitations. First, the validation datasets were relatively small, limiting statistical power and increasing the likelihood of Type II errors, potentially explaining why some predictor genes did not reach significance in validation cohorts. Absence of validation does not preclude their biological relevance, and larger, multi-center studies are needed. Second, while three core genes were identified, their regulatory mechanisms and specific roles in SD-induced neurovascular dysfunction remain incompletely characterized, warranting further *in vitro* and *in vivo* functional studies, as well as additional single-cell analyses. Third, although the NVU-centric model is supported by literature, direct causal relationships between endothelial stress, BBB disruption, and secondary immune remodeling require experimental confirmation through cell-specific genetic manipulations. Fourth, it is important to note that the drug prediction analysis utilized DSigDB, a database that aggregates diverse gene expression signatures. Given the potential noise and heterogeneity in these reference datasets, the identified compounds should be interpreted with caution.

In conclusion, our study demonstrated that SD-induced autophagy dysfunction originates in the neurovascular unit, leveraging bulk and single-cell data. *CDKN1A, HSPA5,* and *NR4A1* serve as specific markers for endothelial and neuronal stress, which collectively shape an immunosuppressive microenvironment. This framework deepens our understanding of SD pathology and informs the development of precise therapeutic interventions.

## Conclusion

*CDKN1A, HSPA5,* and *NR4A1* emerge as potential diagnostic biomarkers for SD with significant clinical utility. This study provides novel molecular targets for elucidating the mechanisms underlying SD-induced autophagy modulation, immune response, and neurovascular injury.

## Supplemental Information

10.7717/peerj.21426/supp-1 Supplemental Information 1 Quality control and preprocessing of the GSE137665 scRNA-seq dataset

10.7717/peerj.21426/supp-2 Supplemental Information 2 Predicted targeted therapeutic drugs based on predictor genes (A) red squares represent miRNAs; blue circles represent lncRNAs; green triangles represent mRNAs. (B) yellow triangles represent mRNAs; sky-blue ellipses represent drugs and their targets.

10.7717/peerj.21426/supp-3 Supplemental Information 3 GSE33302 + GSE9442 DEGs

10.7717/peerj.21426/supp-4 Supplemental Information 4 GSE9441 18 samples DE complete

10.7717/peerj.21426/supp-5 Supplemental Information 5 GSE37667 DEG

10.7717/peerj.21426/supp-6 Supplemental Information 6 MIQE checklist

10.7717/peerj.21426/supp-7 Supplemental Information 7 ARRIVE 2.0 checklist

10.7717/peerj.21426/supp-8 Supplemental Information 8 Supplementary tables Table S1. The primer sequences of qRT-PCR. Table S2. Quantitative analysis of the predictor genes’ expression across experimental groups. Table S3. Independent samples *t*-test results of the predictor genes’ expression between the control and SD groups. Table S4. DSigDB selected predictor gene-associated drugs and targets.

10.7717/peerj.21426/supp-9 Supplemental Information 9 Code for data cleaning & differential analysis

10.7717/peerj.21426/supp-10 Supplemental Information 10 Code for enrichment analysis

10.7717/peerj.21426/supp-11 Supplemental Information 11 Code for machine learning

10.7717/peerj.21426/supp-12 Supplemental Information 12 Code for diagnostic model

10.7717/peerj.21426/supp-13 Supplemental Information 13 Code for immune infiltration

10.7717/peerj.21426/supp-14 Supplemental Information 14 Code for single cell analysis and GSEA analysis

10.7717/peerj.21426/supp-15 Supplemental Information 15 Sleep deprivation effects on mRNA expression in rat hippocampus and prefrontal cortex dataset

## Additional Information and Declarations

### Data Availability

The following information was supplied regarding data availability: All data generated or analyzed in this study were obtained from GEO: GSE33302 and GSE9442; GSE9441, GSE37667; GSE137665. All analysis scripts, including data preprocessing, differential expression analysis, ML pipelines, and visualization routines, are available at GitHub and Zenodo: – https://github.com/tobejoy613/Autophagy-Genes-as-Sleep-Deprivation-Biomarkers. – tobejoy613. (2026). tobejoy613/Autophagy-Genes-as-Sleep-Deprivation-Biomarkers: Initial release for PeerJ publication (v1.0.0). Zenodo. https://doi.org/10.5281/zenodo.20034930.
