# Supplementary figures and images for: Integrated transcriptomic identification and validation reveal key autophagy-associated biomarkers in sleep deprivation
Source: PeerJ. 2026 Jun 3;14:e21426. doi: 10.7717/peerj.21426 (PMC13242190; doi:10.7717/peerj.21426)

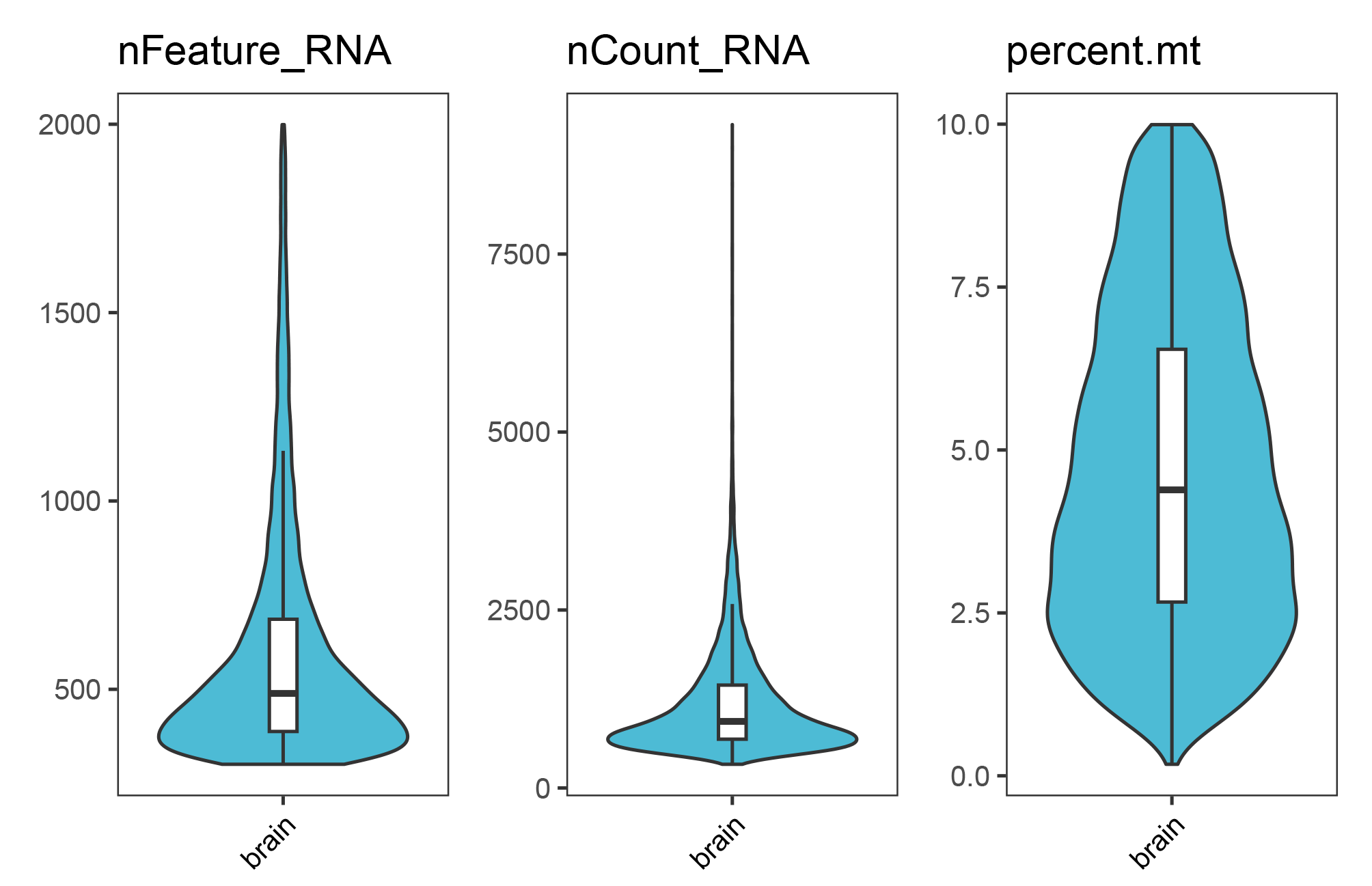

Supplement: Supplemental Information 1 [file peerj-14-21426-s001.png]

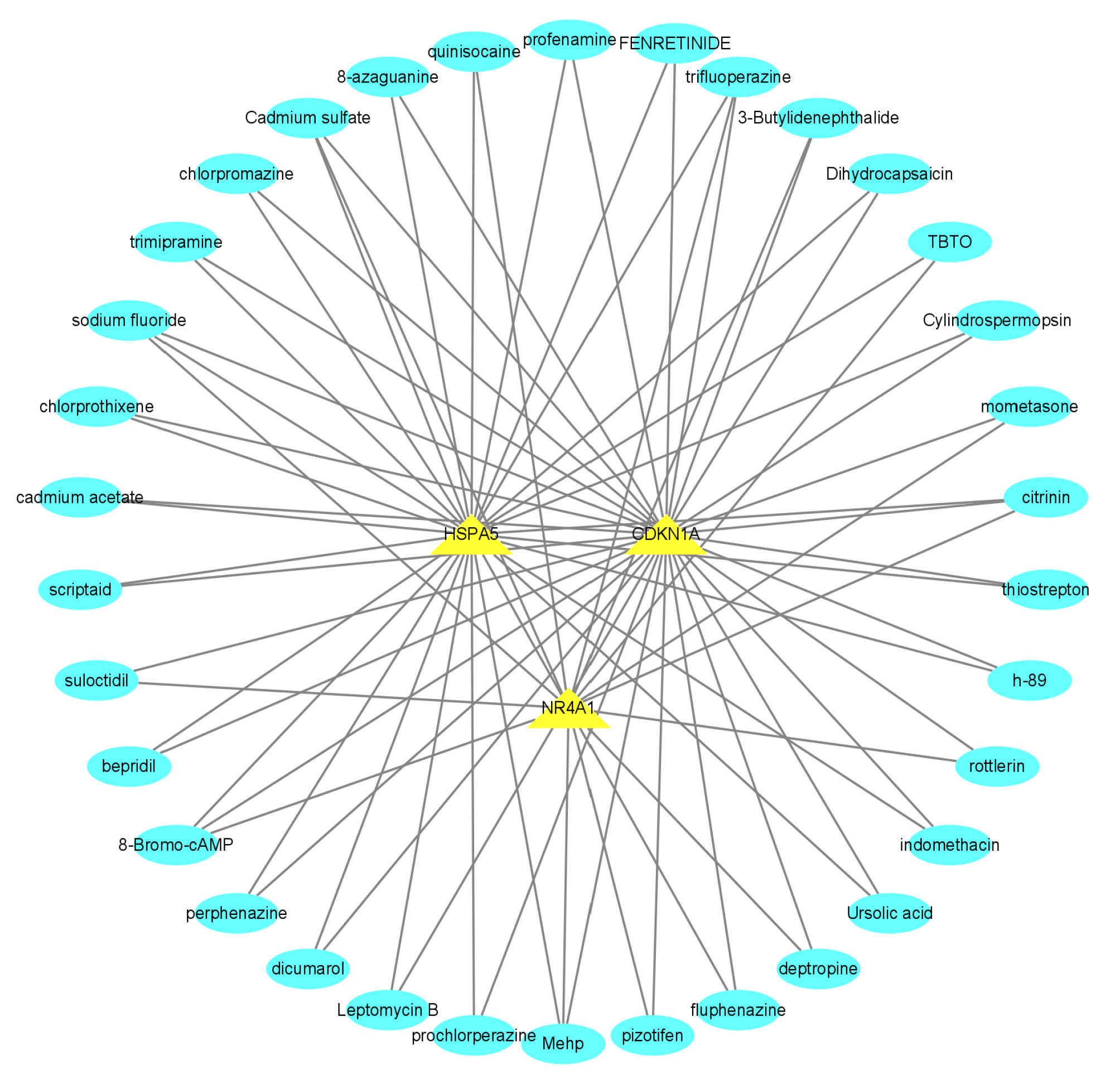

Supplement: Supplemental Information 2 — (A) red squares represent miRNAs; blue circles represent lncRNAs; green triangles represent mRNAs. (B) yellow triangles represent mRNAs; sky-blue ellipses represent drugs and their targets. [file peerj-14-21426-s002.png]
