# Supplementary material for: Integrated transcriptomic identification and validation reveal key autophagy-associated biomarkers in sleep deprivation
Source: PeerJ. 2026 Jun 3;14:e21426. doi: 10.7717/peerj.21426 (PMC13242190; doi:10.7717/peerj.21426)
Supplement: Supplemental Information 8 — Table S1. The primer sequences of qRT-PCR. Table S2. Quantitative analysis of the predictor genes’ expression across experimental groups. Table S3. Independent samples t-test results of the predictor genes’ expression between the control and SD groups. Table S4. DSigDB selected predictor gene-associated drugs and targets. [file peerj-14-21426-s008.docx]

Table S1 The primer sequences of qRT-PCR

| **Primer Name** | **Primer Information** | **Base sequence (5`-3`)** | | **Tm value** | **CG%** | **Product length** |
| --- | --- | --- | --- | --- | --- | --- |
| R-GAPDH | NM_017008.4 | sense | GCCAAGGTCATCCATGACAAC | 59.8 | 52.4 | 152 |
|  |  | antisense | GTGGATGCAGGGATGATGTTC | 60.2 | 61.1 |  |
| R-CDKN1A | NM_080782 | sense | CCCGAGAACGGTGGAACTT | 59.6 | 57.9 | 110 |
|  |  | antisense | CCCAGGGCTCAGGTAGATCTT | 60.7 | 57.1 |  |
| R-HSPA5 | NM_013083 | sense | CCATGCAGTTGTGACTGTACC | 59.2 | 52.4 | 264 |
|  |  | antisense | CCCAGATGAGTGTCTCCATTAG | 57.7 | 50 |  |
| R-NR4A1 | NM_024388 | sense | GCTTCTTCAAGCGCACAGTAC | 60.1 | 52 | 243 |
|  |  | antisense | GAATGAGGGACGTGAGGAGATT | 59.6 | 50 |  |
| R-PRKAB1 | NM_031976 | sense | AGGTCTACTTGTCTGGATCCTTCA | 59 | 45.8 | 114 |
|  |  | antisense | AGAACTTGTACTGATGCTCTCCTTC | 58.4 | 44 |  |

Table S2 Quantitative analysis of the predictor genes’ expression across experimental groups

|  | group | N | Mean | Standard deviation | The standard error of the mean |
| --- | --- | --- | --- | --- | --- |
| CDKN1A | A1.Normal control group | 3 | .7600 | .23516 | .13577 |
|  | B1.Insomnia model group | 3 | 2.4967 | .35346 | .20407 |
| HSPA5 | A1.Normal control group | 3 | 1.1767 | .15948 | .09207 |
|  | B1.Insomnia model group | 3 | 2.2100 | .45826 | .26458 |
| NR4A1 | A1.Normal control group | 3 | .7633 | .26951 | .15560 |
|  | B1.Insomnia model group | 3 | 1.7300 | .16093 | .09292 |
| PRKAB1 | A1.Normal control group | 3 | 1.0500 | .19000 | .10970 |
|  | B1.Insomnia model group | 3 | 1.0100 | .15875 | .09165 |

Table S3 Independent samples t-test results of the predictor genes’ expression between the control and SD groups

|  |  | Levene’s Test |  | T-test |  |  |  |  |  |  |
| --- | --- | --- | --- | --- | --- | --- | --- | --- | --- | --- |
|  |  | F | Sig. | t | df | Sig. (2-tailed) | Mean Difference | Std. Error Difference | CI |  |
|  |  |  |  |  |  |  |  |  | LOWER | UPPER |
| CDKN1A | Equal variances assumed | 1.173 | 0.340 | -7.085 | 4.000 | 0.002 | -1.737 | 0.245 | -2.417 | -1.056 |
|  | Equal variances not assumed |  |  | -7.085 | 3.480 | 0.003 | -1.737 | 0.245 | -2.459 | -1.014 |
| HSPA5 | Equal variances assumed | 2.906 | 0.163 | -3.689 | 4.000 | 0.021 | -1.033 | 0.280 | -1.811 | -0.256 |
|  | Equal variances not assumed |  |  | -3.689 | 2.477 | 0.047 | -1.033 | 0.280 | -2.041 | -0.025 |
| NR4A1 | Equal variances assumed | 0.875 | 0.403 | -5.334 | 4.000 | 0.006 | -0.967 | 0.181 | -1.470 | -0.463 |
|  | Equal variances not assumed |  |  | -5.334 | 3.265 | 0.010 | -0.967 | 0.181 | -1.518 | -0.416 |
| PRKAB1 | Equal variances assumed | 0.117 | 0.750 | 0.280 | 4.000 | 0.793 | 0.040 | 0.143 | -0.357 | 0.437 |
|  | Equal variances not assumed |  |  | 0.280 | 3.877 | 0.794 | 0.040 | 0.143 | -0.362 | 0.442 |
| GAPDH | Equal variances assumed | 0.186 | 0.689 | -2.693 | 3.906 | 0.055 | -0.09667 | 0.0359 | -0.196 | 0.003 |
|  | Equal variances not assumed |  |  | -2.693 |  | 0.056 | -0.09667 | 0.0359 | -0.197 | 0.004 |

Table S4 DSigDB selected predictor gene-associated drugs and targets

| Term | drug | P-value | Adjusted P-value | Old P-value | Old Adjusted P-value | Odds Ratio | Combined Score | Genes |
| --- | --- | --- | --- | --- | --- | --- | --- | --- |
| rottlerin CTD 00003057 | rottlerin | 4.041779821754755E-5 | 8.202435520619943E-4 | 0 | 0 | 553.4722222222222 | 5599.058010484021 | CDKN1A;HSPA5 |
| TBTO CTD 00000264 | TBTO | 3.825055232969356E-5 | 7.997842759845017E-4 | 0 | 0 | 569.3428571428572 | 5790.986926267435 | NR4A1;CDKN1A |
| profenamine PC3 UP | profenamine | 3.825055232969356E-5 | 7.997842759845017E-4 | 0 | 0 | 569.3428571428572 | 5790.986926267435 | CDKN1A;HSPA5 |
| chlorpromazine PC3 UP | chlorpromazine | 3.825055232969356E-5 | 7.997842759845017E-4 | 0 | 0 | 569.3428571428572 | 5790.986926267435 | CDKN1A;HSPA5 |
| chlorprothixene PC3 UP | chlorprothixene | 3.7189269487955056E-5 | 7.997842759845017E-4 | 0 | 0 | 577.6231884057971 | 5891.462103111049 | CDKN1A;HSPA5 |
| scriptaid PC3 UP | scriptaid | 3.6432848434344346E-5 | 7.997842759845017E-4 | 0 | 0 | 58008 | 592844.0664416633 | NR4A1;CDKN1A;HSPA5 |
| 8-Bromo-cAMP, Na CTD 00007044 | 8-Bromo-cAMP | 3.449016231416672E-5 | 7.997842759845017E-4 | 0 | 0 | 58044 | 596392.6054471306 | NR4A1;CDKN1A;HSPA5 |
| Mehp CTD 00000849 | Mehp | 3.4094799344218043E-5 | 7.997842759845017E-4 | 0 | 0 | 603.969696969697 | 6212.653173060806 | NR4A1;CDKN1A |
| deptropine PC3 UP | deptropine | 3.4094799344218043E-5 | 7.997842759845017E-4 | 0 | 0 | 603.969696969697 | 6212.653173060806 | CDKN1A;HSPA5 |
| h-89 CTD 00002586 | h-89 | 3.210631621114883E-5 | 7.997842759845017E-4 | 0 | 0 | 622.90625 | 6444.873216823289 | NR4A1;CDKN1A |
| mometasone PC3 UP | mometasone | 2.6498583919701545E-5 | 7.618342876914194E-4 | 0 | 0 | 687.551724137931 | 7245.708334233761 | CDKN1A;HSPA5 |
| 3-Butylidenephthalide CTD 00001227 | 3-Butylidenephthalide | 2.474861877676981E-5 | 7.424585633030944E-4 | 0 | 0 | 712.1785714285714 | 7553.893565655286 | CDKN1A;HSPA5 |
| quinisocaine PC3 UP | quinisocaine | 2.474861877676981E-5 | 7.424585633030944E-4 | 0 | 0 | 712.1785714285714 | 7553.893565655286 | CDKN1A;HSPA5 |
| trimipramine PC3 UP | trimipramine | 2.1427678773293076E-5 | 7.040523025510581E-4 | 0 | 0 | 767.1153846153846 | 8247.1248435323 | CDKN1A;HSPA5 |
| cadmium acetate CTD 00001325 | cadmium acetate | 2.1427678773293076E-5 | 7.040523025510581E-4 | 0 | 0 | 767.1153846153846 | 8247.1248435323 | NR4A1;CDKN1A |
| suloctidil PC3 UP | suloctidil | 1.985672784850136E-5 | 7.040523025510581E-4 | 0 | 0 | 797.88 | 8638.620967589939 | CDKN1A;HSPA5 |
| perphenazine PC3 UP | perphenazine | 1.8345472263811385E-5 | 7.032431034461031E-4 | 0 | 0 | 831.2083333333334 | 9065.264276038262 | CDKN1A;HSPA5 |
| Leptomycin B CTD 00001805 | Leptomycin B | 1.8345472263811385E-5 | 7.032431034461031E-4 | 0 | 0 | 831.2083333333334 | 9065.264276038262 | NR4A1;CDKN1A |
| pizotifen PC3 UP | pizotifen | 1.6893923989640245E-5 | 7.032431034461031E-4 | 0 | 0 | 867.4347826086956 | 9531.856142980412 | CDKN1A;HSPA5 |
| Ursolic acid CTD 00000232 | Ursolic acid | 1.6190543832870734E-5 | 7.032431034461031E-4 | 0 | 0 | 886.7555555555556 | 9781.874311549509 | CDKN1A;HSPA5 |
| thiostrepton CTD 00006881 | thiostrepton | 1.4169997221564549E-5 | 6.983784344913956E-4 | 0 | 0 | 950.2380952380952 | 10608.82270191872 | CDKN1A;HSPA5 |
| Cylindrospermopsin CTD 00003136 | Cylindrospermopsin | 1.3998726769412396E-5 | 6.983784344913956E-4 | 0 | 0 | 58551 | 654397.838138368 | NR4A1;CDKN1A;HSPA5 |
| trifluoperazine PC3 UP | trifluoperazine | 9.978211166975307E-6 | 5.737471421010801E-4 | 0 | 0 | 1140.6857142857143 | 13135.117740244841 | CDKN1A;HSPA5 |
| 8-azaguanine HL60 UP | 8-azaguanine | 9.732598881573331E-6 | 5.737471421010801E-4 | 0 | 0 | 58716 | 677584.3778513782 | NR4A1;CDKN1A;HSPA5 |
| sodium fluoride CTD 00006755 | sodium fluoride | 7.432436142121351E-6 | 5.128380938063732E-4 | 0 | 0 | 1331.1333333333334 | 15720.22791997129 | CDKN1A;HSPA5 |
| bepridil PC3 UP | bepridil | 6.518791747577496E-6 | 4.997740339809414E-4 | 0 | 0 | 1426.357142857143 | 17031.876058058097 | CDKN1A;HSPA5 |
| dicumarol CTD 00005515 | dicumarol | 6.518791747577496E-6 | 4.997740339809414E-4 | 0 | 0 | 1426.357142857143 | 17031.876058058097 | CDKN1A;HSPA5 |
| prochlorperazine PC3 UP | prochlorperazine | 6.084403864684326E-6 | 4.997740339809414E-4 | 0 | 0 | 1479.2592592592594 | 17765.580935738268 | CDKN1A;HSPA5 |
| fluphenazine PC3 UP | fluphenazine | 5.664974181624268E-6 | 4.997740339809414E-4 | 0 | 0 | 1536.2307692307693 | 18559.523798613252 | CDKN1A;HSPA5 |
| indomethacin CTD 00006147 | indomethacin | 4.657785175310285E-6 | 4.997740339809414E-4 | 0 | 0 | 58995 | 724279.8750650825 | NR4A1;CDKN1A;HSPA5 |
| citrinin CTD 00005677 | citrinin | 3.792250913340651E-6 | 4.997740339809414E-4 | 0 | 0 | 1902.4761904761904 | 23747.755706024196 | CDKN1A;HSPA5 |
| Dihydrocapsaicin CTD 00000675 | Dihydrocapsaicin | 3.1479211460407713E-6 | 4.997740339809414E-4 | 0 | 0 | 2102.9473684210525 | 26641.752908474842 | CDKN1A;HSPA5 |
| FENRETINIDE CTD 00007166 | FENRETINIDE | 1.4622004764763584E-6 | 4.997740339809414E-4 | 0 | 0 | 59316 | 796944.1563059186 | NR4A1;CDKN1A;HSPA5 |
| Cadmium sulfate CTD 00001745 | Cadmium sulfate | 3.6552453980046626E-7 | 2.5221193246232173E-4 | 0 | 0 | 59568 | 882912.870373916 | NR4A1;CDKN1A;HSPA5 |
